# Targeted Child Mental Health Prevention and Parenting Support Within a Canadian Context: A Randomized Controlled Trial Evaluating the U.S.-Developed Family Check-Up®

**DOI:** 10.1007/s11121-024-01741-3

**Published:** 2024-11-22

**Authors:** Teresa Bennett, Katholiki Georgiades, Andrea Gonzalez, Magdalena Janus, Ellen Lipman, Paulo Pires, Heather Prime, Eric Duku, Marc Jambon, John D. McLennan, Julie Gross, the Making the Race Fair Study Team, Sofia Al Balkhi, Krysta Andrews, Erin Baird, Annie Beatty, Amanda Bonomo, Meghan Dovey, Aisha Farooq, Anne Kang, Oya Pakkal, Mandy Sahota, Amy Vanderkooy, Tamara Krbavac, Angie Burroughsford, Melissa Kimber, Andrea Brown, M. Boyle, C. E. Cunningham, N. Kates, C. Waddell, Thomas J. Dishion, David Dan Offord

**Affiliations:** 1 Offord Centre for Child Studies, Department of Psychiatry and Behavioural Neurosciences, McMaster University, Hamilton, ON Canada; 2 Ron Joyce Children’s Health Centre, McMaster Children’s Hospital, Hamilton Health Sciences, Hamilton, ON Canada; 3 Department of Psychology, York University, Toronto, ON Canada; 4 Department of Psychology, Wilfrid Laurier University, Waterloo, ON Canada; 5 Department of Psychiatry and Community Health Sciences, University of Calgary, Calgary, AB Canada

**Keywords:** Parenting, Family check-up®, Prevention, Early childhood mental health, Family-centered care

## Abstract

Canada lacks an approach to early childhood mental health prevention aimed at decreasing barriers to care among highest-needs families. In this Canadian randomized controlled trial, we aimed to evaluate whether participation in the Family Check-Up® (FCU®) would be associated with lower severity of child behavior problems (primary outcome) and caregiver psychological distress and daily parenting stress (secondary outcomes). Eligible caregivers of children aged 2–4 years with (i) high severity of behavior problems and/or (ii) above-average severity plus ≥ 1 family psychosocial risk factor were recruited from early education, community, and clinical settings in Hamilton, Ontario. Randomization: either the FCU® or a community comparison arm (206 analyzed of 207 enrolled). Caregiver reports on their child’s behavior problems (primary outcome, Child Behavior Checklist Externalizing Problems Scale), caregiver psychological distress, and daily parenting stress (secondary) were obtained 12 months after study enrolment, and rates of change were modeled over 0, 6, and 12 months. FCU® participants reported lower child behavior problem severity scores 12 months post-enrolment than did community comparison participants (*d* = 0.38, *p* < 0.01). Caregiver psychological distress (*d* = 0.17, *p* = 0.3) and parenting stress (*d* = .05, *p* = 0.8) did not differ significantly between arms. FCU® participants reported improvements in the severity of child behavior problems, relative to a community comparison group, but not in caregiver distress or parenting stress at 12 months. Positive results for primary outcome indicate the FCU’s® promise as an effective child mental health prevention program in Canada. Further evaluation of intensified caregiver mental health supports may be warranted. Trial registered at Clinicaltrials.gov (#NCT02800603).

**Supplementary Information:**

The online version contains supplementary material available at 10.1007/s11121-024-01741-3.

Severe and persistent early childhood emotional and behavioral problems are arguably the strongest predictors of later mental health problems including depression, conduct problems, and substance use challenges (Angold & Egger, 2007; Luby et al., 2014; Wichstrom et al., 2018). The prevalence of such early difficulties is high among preschool children (12.5–26%) (Egger & Angold, 2006; Wichstrom et al., 2012). The preschool years may represent a particularly critical period for family-centered mental health prevention and early intervention, given very young children’s heightened sensitivity to home and caregiving environments and, particularly, as families recover from the psychosocial and mental health impact of the COVID-19 pandemic (Khoury et al., 2021; Wang et al., 2021). This special issue honors Dr. Robert J. McMahon, whose seminal observational studies, clinical trials, and collaborations have demonstrated that early parenting, family processes, and related interventions may in fact yield long-lasting effects on children’s well-being into adulthood (Dodge et al., 2015; Racz et al., 2019).

Targeted child mental health prevention approaches identify children who experience risk factors and/or early signs of mental health challenges, including aggression and emotional dysregulation. Such programs, often delivered in a group format, typically empower caregivers to support child self-regulation through positive parenting and have demonstrated robust and often sustained improvements in childhood behavior (Dishion et al., 2008; Gardner et al., 2019; van Aar et al., 2017). However, on average, only 50% of caregivers complete parenting programs to which they are referred, with higher dropout rates among caregivers dealing with greater psychosocial adversity such as socioeconomic strain and parental mental illness (Chacko et al., 2016; Reyno & McGrath, 2006). Furthermore, mere attendance is often not enough: It is essential to “work hard” to ensure high-quality engagement, particularly when caregivers face competing stressors (Nix et at., 2009). This reality underscores the need to address underlying social determinants of health within programs that incorporate developmental and ecological (child-within-family-and-community) frameworks and to deliver more flexible, accessible, and collaborative family-centered care (Godwin et al., 2020). However, Canada currently lacks a coherent early childhood mental health framework and a systematic approach to targeted prevention among children at higher risk of severe and persistent child mental health problems (Clinton, 2014).

Actions to begin addressing early mental health in Canada include the implementation and delivery of evidence-based support for caregivers. The Family Check-Up® (FCU®), as developed and evaluated in the United States (U.S.) for families of very young children, may be a particularly good fit (Dishion et al., 2008). The FCU® is a brief, strength-based, and motivational prevention program aimed at improving short- and long-term child and family outcomes for families of children aged 2–18 years. Program developers investigated its effects in two U.S.-based randomized controlled trials (RCTs) with delivery to mothers of 2-year-old children (*n* = 120 (Shaw et al., 2006); *n* = 731 (Dishion et al., 2008)) recruited from Women, Infant and Children (WIC) Nutrition Programs (a U.S.-based program providing nutritional support and healthcare to very low-income families). FCU® participants reported greater improvements in child behavior problems as measured by the Child Behavior Checklist (CBCL, Achenbach & Rescorla, 2001) over a 24-month and 36-month follow-up period (*d* = 0.23, *d* = 0.30) (Dishion et al., 2008, 2014). Studies demonstrated significant improvements in outcomes that included parenting practice at child aged 3 years (*d* = 0.33, Dishion et al., 2008) and teacher-reported behavior problems at 7.5 years (*d* = 0.26) (Dishion et al., 2014). Participation was also associated with small, significant reductions in mothers’ self-reported depression symptoms (*d* = 0.18) which in turn mediated improvements in child outcomes such as conduct problems at age 4 years and internalizing symptoms at age 7.5 years (Reuben et al., 2015; Shaw et al., 2009).

Experts urge caution in assuming that even consistent benefits derived from a complex psychosocial intervention will “hold” in a new country or setting (Catherine et al., 2020; Olds, 2008; Weisz et al., 2020). To date, only one study has evaluated the effectiveness of the FCU® for families of very young children outside of the U.S., in Australia, with null findings; however, major differences in study design preclude drawing conclusions regarding whether findings are related to differences in methodology, population, and setting or program delivery (Hiscock et al., 2018). Other complex, tailored interventions such as the Nurse-Family Partnership and Multi-Systemic Therapy® provide some examples of non-replication and/or differential response patterns in non-U.S. countries, attributed in large part to stronger “treatment as usual” options for control groups in countries with more robust and/or universal health, educational and social safety nets as well as to differences in characteristics of clinical populations enrolled (Littell et al., 2021; Robling et al., 2016). Given the differences in healthcare and populations between Canada and the U.S. and the significant public investments required to scale up complex psychosocial prevention programs, it is essential to evaluate the FCU® as delivered to families of children experiencing early mental health risk factors and compared to a group with access to currently available services for preschool-aged children within a Canadian setting. We aimed, therefore, to conduct a community-based RCT evaluating the FCU® in a sample of caregivers and 2–4-year-old children at high risk for persistent emotional and behavior problems. We hypothesized that FCU® participants would report greater reductions in the severity of child behavior problems (primary outcome) as well as in self-reported caregiver psychological distress and daily parenting stress (secondary outcomes) than would community comparison (CC) participants, 12 months after study enrolment, as well as show greater change of these outcomes from baseline to 12 months.

## Methods

### Recruitment and Screening

The study design was a 1:1 parallel-arm, randomized controlled trial, registered at clinicaltrials.gov (NCT02800603). Approval was obtained from the Hamilton Integrated Research Ethics Board (#1558), and all participating caregivers provided informed consent. Recruitment took place between August 2017 and March 2019 in community settings, through referrals from educators, clinicians, and caregivers themselves, using a strategy prioritizing highest-needs neighborhoods in Hamilton, ON and surrounding regions (Siddiqua et al., 2020; City of Hamilton, n.d.) (see Online Material, Recruitment and Screening). Interested caregivers completed a 10–15-min screening interview with the research coordinator in person or over the telephone. Inclusion criteria were (1) index child aged 2 to 4 years and (2) high caregiver-reported child behavior problems or above-average child behavior problem scores on the 2–4-year-old Strengths and Difficulties Questionnaire (SDQ) (Goodman et al., 2003), plus at least one additional family psychosocial risk factor. Risk factors included (1) caregiver challenges, i.e., teen parent status, moderate to severe caregiver psychological distress (the Kessler-6 [K6] distress scale (Kessler et al., 2003)), and/or lone parent status and (2) sociodemographic risk factors, i.e., family income below the Canadian low-income cut-off (LICO, established annually by Statistics Canada) (Statistics Canada, 2022) and/or primary caregiver with less than grade 12 education. Exclusion criteria included children with suspected severe to profound developmental delay, enrolment in another intervention trial, previous participation in the FCU® (e.g., with another child), a caregiver with insufficient English knowledge to complete assessments, and serious caregiver or child health condition (e.g., acute suicidality) deemed by principal investigators to preclude safe participation.

### Intervention

The FCU® is a brief, one-to-one, flexibly delivered intervention aimed at decreasing child mental health risk by having trained clinicians engage caregivers of children aged 2–18 years in a suite of 3 sessions focused on mindful parenting and goal-setting (Gill et al., 2008). Visits took place in homes or educational or community settings. During the first visit, clinicians establish a collaborative rapport while eliciting caregiver(s) perceptions and goals regarding their child and family context. The second visit involves a comprehensive assessment of child and family risk and protective factors and video-taped caregiver-child interactions. During visit 3, caregiver(s) and clinicians engage in a feedback session based on the assessment data, using motivational interviewing to leverage strengths, support specific positive change, and develop a collaborative menu of services tailored to caregiver goals. In the current study, the menu included a suite of tailored parenting sessions drawn from the “Everyday Parenting Curriculum” (EDP) designed by FCU® developers (maximum of 6) and/or referrals to child or caregiver mental health or community supports (Dishion et al., 2012).

In the current study, FCU® providers reflected the professional backgrounds represented in most Ontario community mental health agencies. Most families (~ 85%) were supported by newly FCU®-trained undergraduate-level clinicians (e.g., Child Life Specialists, Early Childhood Educators), each with over 10 years of parenting intervention experience. The remainder were seen by a Master’s-level social worker or a PhD Psychologist in supervised practice, with assignments based on clinician and caregiver scheduling availability. See Online, Intervention section.

### Randomization and Assessment Schedule

Randomization was computer-generated by the Juravinski Oncology Clinical Operations Group, in concealed blocks, stratified by child sex due to differences in sex-based prevalence of hyperactivity and neurodevelopmental profiles that are often linked to early childhood behavior problems. After a baseline assessment of child, caregiver, and family characteristics, caregivers were informed of their random allocation to either intervention (FCU®) or community comparison (CC) group. FCU® clinicians contacted intervention arm caregivers within 1–2 weeks. Comparison arm caregivers were given a contact list of locally accessible community and mental health agencies providing a range of psychosocial supports, e.g., child mental health, adult counseling, food banks, and recreation, available through self-referral.

Child behavior problems (primary outcome), caregiver psychological stress, and daily parenting stress (secondary outcomes) were measured at baseline (T1) and then at 6 (T2) and 12 months (T3) later. Brief telephone check-ins were conducted at 3 and 9 months for all participants to maintain contact and conduct abbreviated service use questionnaires. Participants in both arms received gift cards for research assessments (not for intervention): $100 for baseline and 12-month visits, $20 for 6-month visits, and $5 for brief 3- and 9-month check-ins. Participating caregivers, study coordinators, intervention providers, and supervisors were, for practical reasons, unblinded to the allocation arm; however, outcome evaluators, assessment staff, and data analysts remained blinded.

### Measures

The primary outcome of interest comprised child behavior problems as measured by the Child Behavior Checklist (CBCL) Externalizing Problems Scale (Achenbach & Rescorla, 2001), a gold standard dimensional primary caregiver-report scale describing their child’s behavior problems (e.g., aggression, tantrums, oppositionality) over the past month using a 3-point scale (0 = *never/not true*, 1 = *somewhat/sometimes true*, 2 = *very or always true*). Higher scores reflect more severe problems. Summed raw scores were used to model growth, with *t*-scores used to describe child scores in relation to population norms: 65 + = clinically severe behavior problems, 60 to 64 = borderline levels of severity, and < 60 = normative/not clinically severe.

Secondary outcomes comprised caregiver psychological distress measured using the Kessler-6 Distress Scale (Kessler et al., 2003) and parenting stress using the Parenting Daily Hassles Scale (Crnic & Booth, 1991). The K6 is a 6-item primary caregiver self-report screening questionnaire measuring anxiety and depression symptoms. Scores of 8–12 have been shown to index moderate depressive symptoms, and 13 + indicates severe levels of depression among Canadian adults (Chiu et al., 2017; Vigod et al., 2011). The Parenting Daily Hassles Scale (Crnic & Booth, 1991; Crnic & Greenberg, 1990) is a primary caregiver-report, frequency scale: 20 items describe discrete events involving regularly occurring, potentially hassling child behavior or events (e.g., “the kids are hard to manage in public”) or parenting tasks (e.g., “continually cleaning up messes of toys or food” with 4 frequency score options including “rarely, sometimes, a lot, constantly.” See Online, Measures section, for descriptions of sociodemographic and screening measures).

### Analytic Plan

Sample size analyses estimated a required sample size of *n* = 210 for the primary outcome based on power analyses estimating a low-moderate effect size of Cohen’s *d* = 0.35 at 12 months, power of 0.80, level of significance 0.05, and wave-to-wave correlation of 0.76 between assessments using RMASS2 software (Hedeker et al., 1999). Conservatively estimating an attrition rate of 25% over 12 months yielded an initial recruitment target sample size of *n* = 280 (Hedeker et al., 1999).

We conducted analyses using an intention-to-treat approach with M*plus* 8.5 using the maximum likelihood robust estimator (MLR). First, to test the primary hypothesis that children in the FCU®, relative to comparison participants, would evidence lower behavior problem scores and caregivers would report lower caregiver distress and daily parenting stress scores (secondary outcomes) at the 12-month follow-up, mean comparisons were conducted using multigroup modeling. We then tested the effect of FCU® intervention on changes in primary and secondary outcomes using latent growth curve modeling (LGCM). We first estimated linear and piecewise LGCMs without predictors (unconditional models) to determine the growth function that best characterized the data. Recommended criteria were used to evaluate the fit of the linear model (Little, 2013) (see Online, Analytic Plan and Results for full details including handling of missing data). We then tested a model with predictors included (conditional model) by regressing the slope factor(s) onto the dummy-coded treatment status variable (0 = comparison, 1 = FCU®).

We included baseline level of caregiver-reported daily parenting stress as a covariate throughout intervention-related analyses, as it was the only variable found to be significantly different between groups at baseline, as well as child sex (stratification variable). In addition to computing unstandardized regression coefficients reflecting differences between FCU® and comparison on changes in outcomes, we also calculated Cohen’s *d* effect sizes to describe the magnitude of the standardized mean differences between the treatment and control groups. In the growth models, we report effect sizes for differences in interval-to-interval change (i.e., over 6 months) as well as the overall efficacy of the treatment (i.e., over the full 12 months of the study).

The 12-month follow-up visits for the final 26 families (March–June 2020) were conducted during the COVID-19 pandemic–related lockdown period, when Canadian social distancing rules barred in-person assessments and children were not permitted to attend a school or early childcare. To evaluate the potential impact of such restrictions on child and family well-being and on study outcomes, we compared mean 12-month outcomes between caregivers who participated before and during the lockdown and performed two types of sensitivity analyses: first, treating data from pandemic lockdown assessments as “missing” and, second, covarying for timing of assessment (pre-/during lockdown) in evaluation analyses.

## Results

### Sample Description and Recruitment

**Fig. 1:**
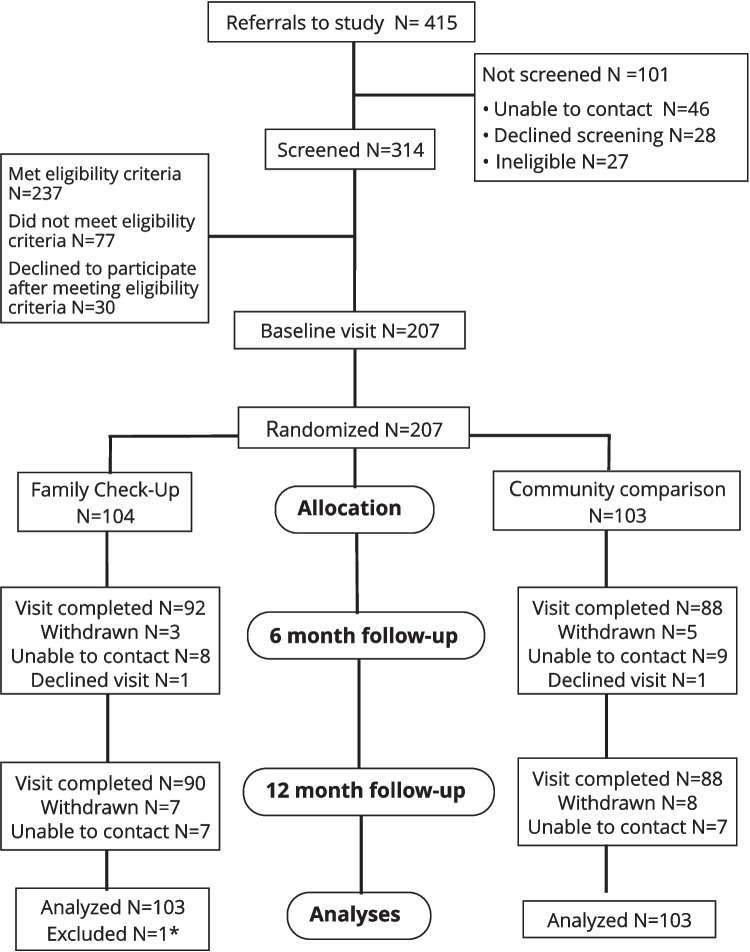
CONSORT flow diagram. *One caregiver-child dyad excluded from the final analyses because the same child was randomized twice due to changes in caregiver custody. See Results Section, Supplementary Materials for details

Caregiver-child dyads were referred for screening through outreach to community settings (e.g., caregiver-child drop-in centers, shelters, *n* = 130, 31.3%) and early childcare and education settings (*n* = 122, 29.4%), self-referral (e.g., from social media, bus advertisements, *n* = 75, 18.1%), clinician referral (e.g., pediatricians, speech-language pathologists, *n* = 29, 7%), and word of mouth (*n* = 32, 7.7%, see Fig. 1). At baseline, 46% (*n* = 94) of the children met criteria for clinically severe behavior problems as indexed by the CBCL and 38% (*n* = 78) of caregivers self-reported moderate to severe levels of distress on the K6. See Table 1 for the description of the sample and Online, Table 2 for bivariate correlation analyses.

**Table 1:** Baseline characteristics by group assignment

Characteristics	FCU®* (*n* = 103)	Community comparison (*n* = 103)	*p* value
Child age in months, mean (SD)	40.63 (10.1)	41.44 (10.8)	0.69
Child sex at birth			0.56
Female	44 (42.7%)	44 (42.7%)	
Male	59 (57.3%)	59 (57.3%)	
Primary caregiver relationship to child			0.46
Biological parent	98 (95.1%)	101 (98.1%)	
Other	5 (4.9%)	2 (1.9%)	
Primary caregiver age in years, mean (SD)	33.52 (5.4)	33.76 (5.6)	0.47
Primary caregiver self-reported racial and/or ethnic identity **	*n* = 103	*n* = 103	0.50
White	90 (87.4%)	92 (89.3%)	
Visible minority or Indigenous	13 (12.6%)	11 (10.7%)	
Primary caregiver marital status	*n* = 101	*n* = 102	0.61
Married/common-law	72 (71.3%)	74 (72.5%)	
Other	29 (28.7%)	28 (27.5%)	
Data missing/prefer not to answer	2 (1.9%)	1 (1.0%)	
Primary caregiver’s highest level of education	*n* = 102	*n* = 103	0.87
Completed up to high school	25 (24.5%)	22 (21.4%)	
Completed up to college	37 (36.3%)	39 (37.9%)	
University +	40 (39.2%)	42 (40.8%)	
Data missing/prefer not to answer	1 (1.0%)		
Household income	*n* = 77	*n* = 81	0.34
Below Canadian low-income cut-off (LICO)	32 (31.1%)	27 (35.1%)	
At/above LICO	45 (43.7%)	54 (52.4%)	
Data missing/prefer not to answer	26 (25.2%)	22 (21.4%)	

All estimates are observed

^*^Family Check-Up®. **Racial and ethnic categories endorsed by primary caregivers as outlined in 2011 Statistics Canada National Household Survey and Visible Minority/Population Groups questionnaires and include Aboriginal, Arab, Black, Chinese, Filipino, Latin American/Hispanic, and Southeast Asian. Individual breakdown of self-reported identification as Indigenous and/or member of specific visible minority groups was not possible due to small cell size of responses

Study recruitment was stopped at *n* = 207 (*n* = 104 FCU® arm; *n* = 103 comparison) in June 2019 due to time and funding constraints. Of those randomized to the FCU®, 97.1% (*n* = 100) completed the full FCU®, 86.5% (*n* = 89) additionally participated in at least one optional EDP session, and 44.7% (*n* = 46) completed the maximum of six (mean = 4.1, SD = 2.20; median = 5). Study follow-up visits ended in June 2020, with 26 assessments conducted over the telephone rather than in person due to COVID-19 restrictions. One caregiver-child dyad was excluded from final analyses because the same child was randomized twice due to changes in caregiver custody with parents enrolling separately at different times during the study. Participants completed primary and secondary outcome questionnaires on iPad during in-person visits and through email during the pandemic lockdown.

### Attrition and Observed Primary Outcome Scores over Time

Complete data (i.e., all study visits completed) were provided by 176/206 participants (85.4%). Study attrition did not differ by intervention arm status but was higher among families with a younger caregiver, lower levels of education, and lower household income (Table 1, Online). This suggested that longitudinal attrition was partly accounted for by study variables and, as such, could reasonably be handled under a missing-at-random (MAR) assumption using full-information maximum likelihood (FIML) according to best practice principles (Little, 2013). We therefore retained the full sample for all analyses (*N* = 206) and included correlates of missingness listed above as auxiliary variables in all estimated models (Rubin, 1976).

### Mean Comparisons (Primary Analysis)

Observed outcome scores across time points are summarized in Table 2. Multigroup models comparing outcomes at baseline revealed no statistically significant group differences for child behavior problems (*M*_FCU_ = 25.04, SD_FCU_ = 9.24; *M*_CC_ = 25.77, SD_CC_ = 8.59; *M*_diff_ = 0.73, *p* = 0.56,* d* = 0.08) or caregiver distress (*M*_FCU_ = 7.85, SD_FCU_ = 4.96; *M*_CC_ = 7.79, SD_CC_ = 4.69; *M*_diff_ = 0.07, *p* = 0.92, *d* = 0.01), but caregivers in the FCU® condition had significantly lower daily hassle scores than did caregivers in the comparison condition at baseline (*M*_FCU_ = 2.45, SD_FCU_ = 0.53; *M*_CC_ = 2.61, SD_CC_ = 0.52; *M*_diff_ = 0.16, *p* = 0.03, *d* = 0.30). At 12-month follow-up, FCU® children had significantly lower behavior scores than comparison children (*M*_FCU_ = 17.68, SD_FCU_ = 10.08; *M*_CC_ = 21.66, SD_CC_ = 9.16; *M*_diff_ = 3.98, *p* = 0.005, *d* = 0.41). To provide clinical context, the baseline average score for both FCU® and comparison groups was at the cut-off for clinically severe behavior problems as indexed by the CBCL Externalizing Problems (*t*-score = 65). Twelve months later, the mean score of FCU® participants’ children declined to the “not clinically at risk” range (a change of 8.7 points or 0.9 SD on the CBCL Externalizing Problems, *t*-score = 55.9), while comparison participant mean score declined to a value designating “at-risk” of clinically severe problems (*t*-score change of 5.4 points from 65.4 to 60.0). No significant differences between the FCU® intervention and comparison arms were found for 12-month caregiver distress (*M*_FCU_ = 6.77, SD_FCU_ = 4.63; *M*_CC_ = 7.35, SD_CC_ = 4.57; *M*_diff_ = 0.58, *p* = 0.40, *d* = 0.13). Caregivers in the FCU® condition had marginally lower 12-month daily hassle scores (*M*_FCU_ = 2.29, SD_FCU_ = 0.55; *M*_CC_ = 2.43, SD_CC_ = 0.56; *M*_diff_ = 0.14, *p* = 0.08, *d* = 0.26).

**Table 2:** Observed scores for child and caregiver outcomes at baseline and follow-up assessments

	FCU®			Community comparison		
	*n*	*M*	SD	*n*	*M*	SD
Baseline behavior problems (raw scores)*	103	25.04	9.3	103	25.8	8.6
6-month behavior problems (raw scores)	92	18.5	9.9	86	20.6	8.2
12-month behavior problems (raw scores)	89	17.7	10.2	87	21.2	9.0
Baseline behavior problems (*t*-scores)	103	64.6	11.0	103	65.4	10.0
6-month behavior problems score (*t*-scores)	92	56.9	12.0	86	59.4	9.4
12-month behavior problems (*t*-scores)	89	55.9	12.1	87	60.0	10.4
Baseline caregiver distress**	103	7.9	5.0	103	7.8	4.7
6-month caregiver distress	92	6.5	4.0	86	7.0	4.5
12-month caregiver distress	90	6.7	4.7	87	6.8	4.3
Baseline daily parenting stress (mean score)†	85	2.4	0.5	83	2.6	0.5
6-month daily parenting stress (mean score)	85	2.2	0.5	83	2.4	0.5
12-month parenting stress (mean score)	85	2.3	0.5	83	2.4	0.5

^*^As measured using the Child Behavior Checklist (CBCL) Externalizing Problems Scale, range of observable summed raw scores, 0–48, and *t*-scores, 28–100. **As measured using the Kessler-6 (K6) Psychological Distress Scale, range of observable summed scores 0–24

^†^As measured using the Parenting Daily Hassles score, range of potential observable mean scores 0–4. For all scales, higher scores represent greater symptom severity

### Growth Models

#### Child Behavior Problems

**Fig. 2:**
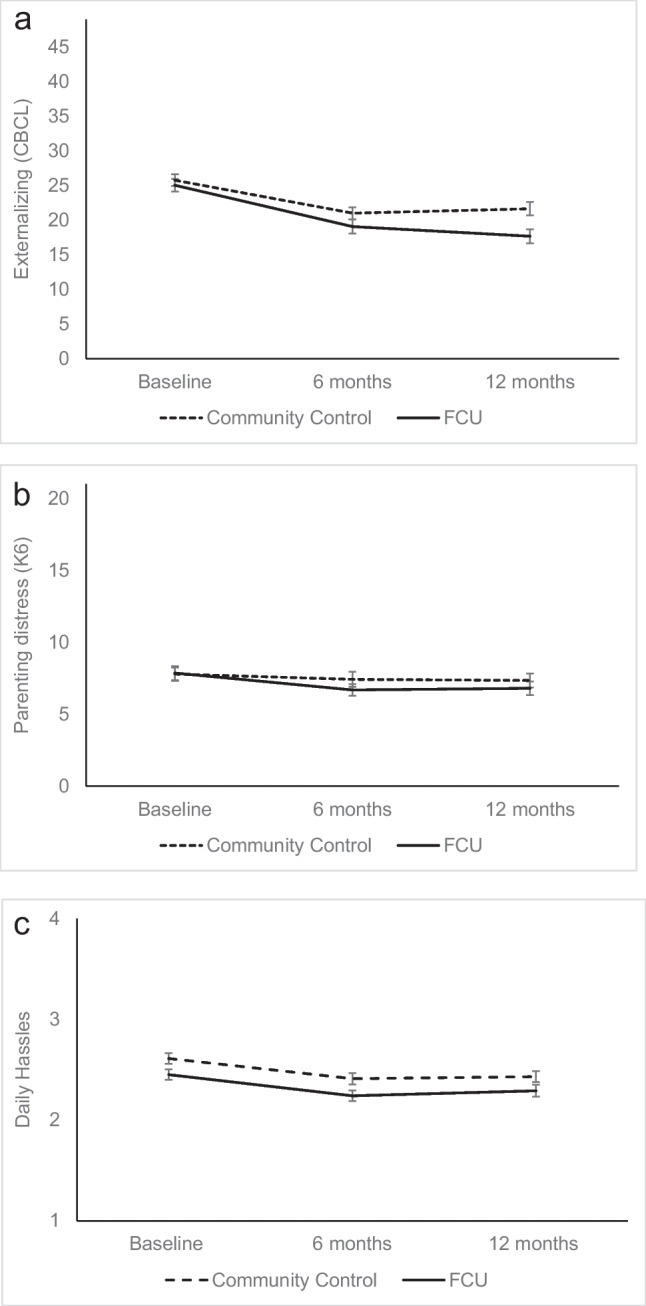
**a** Observed means for caregiver-reported child behavior problems, Child Behavior Checklist (CBCL) Externalizing Problems Scale at baseline and 6 and 12 months post-enrolment. **b** Observed means for caregiver-reported psychological distress, Kessler-6 (K6) at baseline and 6 and 12 months post-enrolment. **c** Observed means for caregiver-reported Daily Parenting Stress measured using the Parenting Daily Hassles Scale at baseline and 6 and 12 months post-enrolment

The linear LGCM provided a poor fit to the data. Estimates from the piecewise growth model indicated that rates of change in behavior problems differed from baseline and 6-month follow-up and 6-month to 12-month follow-up (see Table 3 and Fig. 2a and Results Section, Online, for more details). Average levels of child behavior problems declined from baseline to 6-month follow-up (slope 1), whereas no significant mean level change was observed between 6- and 12-month follow-ups (slope 2) in the entire study sample. We then tested a conditional model with intervention conditions, daily parenting stress, and child sex included as predictors of behavior problem slopes. A model with the predictor effects constrained to equality across the two slopes provided an excellent fit to the data, and allowing the intervention effects to differ across the slopes did not significantly improve the model fit. Children of FCU® participants showed faster declines in the behavior problems (between-group difference in interval-to-interval change *d* = 0.19; overall treatment efficacy* d* = 0.38). From baseline to 6 months, the comparison group declined by 4.5 raw score units, on average, on the CBCL Externalizing Problems Scale, whereas the FCU® group declined by 6.2 units. From 6 to 12 months, the CBCL score increased in the comparison arm by 0.4 raw score units, whereas the CBCL scores in the FCU® group continued to decline by 1.33 units.

**Table 3:** Growth curve model estimates

	Child behavior problems				Caregiver distress				Daily parenting stress			
	*M*	SD	*p* ≤ of mean	95% CI of mean	*M*	SD	*p* ≤ of mean	95% CI of mean	M	SD	*p* ≤ of mean	95% CI of mean
Unconditional models												
Intercept (baseline)	25.4	8.9	.001	24.2, 26.4	7.7	4.2	.001	7.1, 8.4	2.5	0.5	.001	2.5, 2.6
Slope 1	− 5.3	7.0	.001	− 6.4, − 4.3	− 0.4	1.4	.009	− 0.7, − 0.1	− 0.2	0.4	.001	− 0.3, − 0.1
Slope 2	− 0.5	6.5	.4	− 1.5, 0.5	–	–	–	–	0.03	0.4	.4	− 0.03, 0.09
	Estimate	SE	*p* ≤	95% CI	Estimate	SE	*p* ≤	95% CI	Estimate	SE	*p* ≤	95% CI
Conditional models												
Slope 1 intercept	− 4.5	0.6	.001	− 5.6, − 3.3	− 0.3	0.2	.002	− 0.5, 0.1	− 0.2	.04	.001	− 0.3, − 0.2
Slope 2 intercept	0.4	0.6	.6	− 0.8, 1.4	–	–	–	–	0.02	.03	.6	− 0.05, 0.09
Condition slope	− 1.7	0.6	.003	− 2.8, − 0.6	− 0.4	0.3	.3	− 1.2, 0.3	0.01	.03	.8	− 0.06, 0.07
Child sex slope	0.2	0.6	.8	− 0.9, 1.3	0.1	0.3	.9	− 0.5, 0.6	0.03	.03	.3	− 0.03, 0.10
T1 daily parenting stress slope	− 0.7	0.6	.2	− 1.8, 0.4	− 0.3	0.4	.3	− 1.0, 0.3	–	–	–	–

Estimates for predictors represent unstandardized regression coefficients. Child behavior problems as measured using the Child Behavior Checklist Externalizing Problems Scale. Caregiver distress as measured using the Kessler-6 Psychological Distress Score. Daily parenting stress as measured using the Parenting Daily Hassles Scale

#### Caregiver Psychological Distress and Daily Parenting Stress

Average levels of caregiver distress declined linearly over time. In the conditional model, caregivers receiving the FCU® did not decline at a significantly different rate from comparison caregivers (between-group difference in interval-to-interval change *d* = 0.08; overall treatment efficacy* d* = 0.17, Fig. 2b). The unconditional linear model for parenting stress provided a poor fit to the data. Estimates based on the piecewise model indicated that daily parenting stress declined from baseline to 6-month follow-up, whereas no significant mean level decline was observed between the 6- and 12-month follow-ups. Rates of change in daily parenting stress did not differ between caregivers in the FCU® and CC groups (interval-to-interval change *d* = 0.02; overall treatment efficacy* d* = 0.05, Fig. 2c).

#### Sensitivity Analyses

See Table 4 for a descriptive comparison of outcome scores before vs. during the pandemic lockdown. No differences were found for reports of child behavior problems or daily parenting stress, and sensitivity analyses for growth curve models revealed no significant differences in estimates of change in these outcomes when adjusting for assessments conducted before vs. during the COVID-19 lockdown. However, caregiver psychological distress symptoms 12 months post-enrolment were significantly higher among caregivers whose visits took place during the lockdown period compared to the rest of the sample. Sensitivity analyses in which timing of assessment (before/during the pandemic period) was included as a covariate in growth curve analyses indicated a trend toward a greater decline in caregiver distress symptoms in the FCU® group compared to CC (overall treatment efficacy *d* = 0.21, *p* = 0.09, see Online, Table 5).

**Table 4:** Observed descriptives for child and caregiver outcomes for families that completed the 12-month follow-up assessment before and during the COVID-19 pandemic

	Before			During		
	*n*	*M*	SD	*n*	*M*	SD
Baseline behavior problems (raw scores)*	151	25.26	8.64	26	25.38	10.72
6-Month behavior problems (raw scores)	146	19.54	8.97	23	19.04	9.92
12-Month behavior problems (raw scores)	151	19.06	9.58	25	21.60	10.71
Baseline behavior problems (*t*-score)	151	64.80	10.00	26	65.04	12.84
6-month behavior problems (*t*-score)	146	58.03	10.67	23	57.61	11.76
12-month behavior problems (*t*-score)	151	57.50	11.23	25	60.52	12.61
Baseline caregiver distress	151	7.54	4.52	26	7.31	4.87
6-month caregiver distress	146	6.79	4.34	23	6.17	3.46
12-month caregiver distress	151	6.61	4.55	26	7.46	4.00
Baseline parenting stress	151	2.53	0.52	26	2.54	0.63
6-month parenting stress	146	2.31	0.53	23	2.28	0.55
12-month parenting stress	151	2.36	0.55	26	2.34	0.64

^*^Child behavior problems, as measured using the Child Behavior Checklist (CBCL) Externalizing Problems Scale, range of observable summed raw scores, 0–48, and *t*-scores, 28–100. **As measured using the Kessler-6 (K6) Psychological Distress Scale, range of observable summed scores 0–24

^†^As measured using the Parenting Daily Hassles score, range of potential observable mean scores 0–4. For all scales, higher scores represent greater symptom severity

## Discussion

We conducted a community-based RCT of the FCU® as delivered to Canadian caregivers of children showing early signs and risk factors for later emotional and behavioral problems. Caregivers in the FCU® arm rated their children as having significantly fewer behavior problems 12 months after study enrolment than did a community comparison group. On average, FCU® participants also reported significant and persistent declines in their children’s behavior problem scores over the 12-month period, from a score indicative of clinically severe aggression, non-compliance, and meltdowns to one within the normative range of a gold standard parent-report questionnaire. In contrast, community comparison group caregivers reported significantly smaller declines in their child’s behavior problem scores during the first 6-month period, after which scores plateaued. This supports theory and evidence that changes in child behavior and emotional self-regulation can take time, as children adjust to shifts in parenting and family interactions (Dishion et al., 2008). There were small, non-significant 12-month differences in caregiver self-reports of psychological distress. No differences were found in daily parenting stress after accounting for baseline differences between intervention and comparison groups. Taken together, these findings indicate that the FCU® as delivered within a Canadian community setting by providers representative of those typically employed in community mental health agencies was associated with significant and clinically meaningful reductions in early childhood behavior problems. However, further innovation may be required to address the mental health challenges and daily stress of parents and caregivers more effectively.

These results corroborate and extend findings from previous U.S. studies and underscore the importance of careful testing of complex psychosocial prevention programs, including their re-evaluation when establishing them in new countries (Nix et al., 2009). In U.S. studies, participants were drawn from a high-needs population defined by eligibility for Medicaid and nutritional support (Dishion et al., 2008). By contrast, Canadian study participants had access to a healthcare system that, despite being universal, nevertheless lacks a coherent strategy for identifying high-needs preschoolers, which necessitated a broad, mixed approach to recruitment and screening. Participants in our study reported rates of caregiver distress and family income strain that were considerably higher than Canadian averages (Chiu et al., 2017); however, the number of families living in poverty was comparatively lower than the U.S. Early Steps Study in which two-thirds of households lived on less than twenty thousand dollars per year (e.g., Dishion et al., 2008). Such differences in recruitment strategies and participant socio-demographics may dilute the potential benefits of complex psychosocial interventions (Littell et al., 2021; Sundell et al., 2008). However, the effect sizes for primary child behavior problem scores in our study were similar in magnitude to U.S. early childhood FCU® studies (Dishion et al., 2008; Shaw et al., 2006) and meta-analyses of other, often more intensive, evidence-based parenting and child mental health prevention programs (Gardner et al., 2019; Leijten et al., 2019).

We did not find significant differences in caregiver distress related to participation in the FCU®. The current study may have been underpowered to detect small changes in this construct. Caregivers who were assessed during the pandemic lockdown period also reported more severe depressive symptoms compared to those assessed prior to the start of this period. Covarying for assessment timing in relation to the pandemic yielded a relatively stronger, albeit still small, effect size (*d* = 0.25) that trended toward significance. The effect size obtained for caregiver distress aligns with those of the larger U.S. Early Steps Study, which found small but significant effect sizes for caregiver depression (Reuben et al., 2015; Shaw et al., 2009). Furthermore, even small effects on maternal depression may mediate important longer-term downstream differences in child mental health. For example, a recent, large integrated data analysis of 3 FCU® studies (including 2 studies of the program as delivered to preadolescent children) demonstrated a reduction in depressive symptoms among children whose caregivers participated in the FCU®, which was sustained for 10 years and mediated by early improvements in maternal depression (Connell et al., 2021).

The strengths of our study include a RCT design, low study attrition rates, and relatively high rates of caregiver engagement in intervention, with over 85% of FCU® participants engaging in at least one additional follow-up session. Future research stemming from the current study should build on the legacy of Dr. McMahon, whose work underscored the importance of careful attention to the clinical and ethical aspects of outreach, engagement, and equity in a manner that permits the replication and sustainability of targeted prevention programs (McMahon et al., 2021; Nix et al., 2009). This includes future investigation of child and caregiver profiles of “responders vs. non-responders” using data from the current study through latent profile analyses. Next-step Family Check-Up® implementation studies should involve investigating optimal “clinical homes” for the FCU® in Canada, which may include evaluation of universally delivered screening and referral pathways within universal healthcare or early education settings. Integrated co-design partnerships may help optimize caregiver engagement and minimize stigma related to the screening and identification of children at higher mental health risk, particularly among more marginalized groups. To enhance caregiver mental health benefits, future extensions of the current study may include intensifying or adapting integrated caregiver mental health and psychosocial services supports linked to the FCU® within child mental health or pediatric settings, expediting parent/caregiver access to adult mental healthcare services, and ensuring high-quality psychosocial service supports and service navigation that address the daily living stresses of high-needs families.

###  Limitations

Recruitment efforts failed to yield the anticipated sample size, which might have further strengthened the reliability of estimates. We relied on caregiver reports of child behavior: it is possible that changes reported by unblinded caregivers in the intervention arm may have inflated true differences between groups. Another limitation was the relative racial and ethnic homogeneity of our sample. American studies have shown the FCU® to be effective across different U.S. population groups (Smith et al., 2014); further implementation research will be required in partnership with Canada’s diverse communities. Finally, our recruitment strategy and use of financial incentives may have resulted in the recruitment of a more motivated participant group. Research, albeit mixed, does suggest that financial incentives may optimize recruitment rates (Gross & Bettencourt, 2019; Heinrichs, 2006; Highlander et al., 2021), but not necessarily outcomes (Heinrichs & Jensen-Doss, 2010; Highlander et al., 2021) of parenting programs. In contrast, the effect sizes for parenting programs are consistently larger for clinically referred samples than in prevention studies (Gardner et al., 2019; Leijten et al., 2019; van Aar et al., 2017), which supports the generalizability of the current study’s findings to clinical settings. Future research may investigate whether incentivizing family-centered programs is a cost-effective Canadian public health strategy.

## Conclusion

We found evidence to support the FCU® as a targeted prevention intervention that is new to the Canadian child mental health services landscape with clinically meaningful effects for child behavior problems, but less clearly for caregiver distress and not for daily parenting stress. This program may be effectively and flexibly delivered by supervised teams of allied health professionals across a range of community, primary care, or mental health settings. Its brevity and comprehensive motivational approach emphasizing social determinants of child and family well-being may fill a gap in “upstream” child mental health prevention services. We congratulate Dr. McMahon, a true leader in the field of prevention and Professor at Canada’s Simon Fraser University, on his retirement, with thanks for his foundational efforts to improve the lives of children and families everywhere.

## Supplementary Information

Below is the link to the electronic supplementary material.

Supplementary file1 (DOCX 87.9 KB)

## Data Availability

Data is available upon request from the corresponding author.
