# Correction to: Targeted Child Mental Health Prevention and Parenting Support Within a Canadian Context: A Randomized Controlled Trial Evaluating the U.S.-Developed Family Check-Up®

**DOI:** 10.1007/s11121-024-01764-w

**Published:** 2024-12-27

**Authors:** Teresa Bennett, Katholiki Georgiades, Andrea Gonzalez, Magdalena Janus, Ellen Lipman, Paulo Pires, Heather Prime, Eric Duku, Marc Jambon, John D. McLennan, Julie Gross, the Making the Race Fair Study Team

**Affiliations:** 1 Offord Centre for Child Studies, Department of Psychiatry and Behavioural Neurosciences, McMaster University, Hamilton, ON Canada; 2 Ron Joyce Children’s Health Centre, McMaster Children’s Hospital, Hamilton Health Sciences, Hamilton, ON Canada; 3 Department of Psychology, York University, Toronto, ON Canada; 4 Department of Psychology, Wilfrid Laurier University, Waterloo, ON Canada; 5 Department of Psychiatry and Community Health Sciences, University of Calgary, Calgary, AB Canada

**Correction to: Prevention Science**

**https://doi.org/10.1007/s11121-024-01741-3**

In the original version of this article, the given and family names of all authors were incorrectly structured. The names were displayed correctly in all versions at the time of publication.

In the published version, they were captured as:

Bennett Teresa A., Georgiades Katholiki, Gonzalez Andrea, Janus Magdalena, Lipman Ellen, Pires Paulo, Prime Heather, Duku Eric, Jambon Marc, McLennan John D., Gross Julie, the Making the Race Fair Study Team

This should be corrected to:

Teresa Bennett, Katholiki Georgiades, Andrea Gonzalez, Magdalena Janus, Ellen Lipman, Paulo Pires, Heather Prime, Eric Duku, Marc Jambon, John D. McLennan, Julie Gross, the Making the Race Fair Study Team

The original article has been corrected.

