# Supplementary material for: Targeted Child Mental Health Prevention and Parenting Support Within a Canadian Context: A Randomized Controlled Trial Evaluating the U.S.-Developed Family Check-Up®
Source: Prev Sci. 2024 Nov 22;26(4):555–67. doi: 10.1007/s11121-024-01741-3 (PMC12208979; doi:10.1007/s11121-024-01741-3)
Supplement: Supplementary file 1 — Supplementary file1 (DOCX 87.9 KB) [file 11121_2024_1741_MOESM1_ESM.docx]

Supplementary Online Materials

METHODS

Study Design: Study design was a 1:1 parallel-arm, intention to treat randomized controlled trial, registered at clinicaltrials.gov (#NCT02800603). Approval was obtained from the Hamilton Integrated Research Ethics Board (HiREB). The study design, oversight, analysis and write-up were conducted independently of developers of the Family Check-Up^®^ (FCU^®^, Dr. Thomas Dishion, REACH/Arizona State University, and colleagues). FCU^®^ developers trained the study clinical team and supervisors.

Recruitment: The recruitment of families occurred between August 2017 and March 2019. Our recruitment outreach strategy prioritized highest-needs neighbourhoods in Hamilton, ON and surrounding regions, as characterized by high rates (i.e., 41.6%-52.2%) of childhood vulnerability on the Early Development Instrument (EDI), an index of developmental health that is highly correlated with neighbourhood socioeconomic status^1, 2^. Families were recruited from childcare settings, schools, EarlyON Centres (drop-in centres supporting caregivers of young children), low-income housing developments, libraries, community organizations and primary care practices as well as through advertisements posted on parent-focused social media sites and in Hamilton City buses.

Screening: Interested caregivers completed a 10-15 minute screening interview with the research coordinator in person or over telephone. Families were included if: a) caregiver-reported emotional and behaviour problem (behaviour problems) scores fell within the “high” or “very high” range on the total Strengths and Difficulties Questionnaire (SDQ >=16 ) or conduct (>=5) , hyperactivity (>=7) or peer difficulties (>=4) subscales, OR b) above-population-average scores on the total SDQ (13-15), or SDQ conduct (=4), hyperactivity (=6) or peer difficulties subscales (=3) AND at least one family psychosocial risk factor for childhood mental health problems: i) Caregiver challenges: teen parent status, caregiver psychological distress (Kessler 6 score >=12), lone parent; (ii) Sociodemographic risk factors: family income below low-income cut-off [LICO], primary caregiver with less than grade 12 education. Exclusion criteria included children with suspected severe to profound developmental delay, enrolment in another clinical intervention trial, caregiver with insufficient English knowledge to complete study assessments, and caregiver or child with a serious health condition (e.g., acute suicidality) precluding safe participation.

Intervention: The Family Check-Up^®^ (FCU^®^) is a brief, flexibly delivered intervention aimed at decreasing child mental health risk by engaging caregivers of children aged 2-18 years in a suite of 3 sessions focused on mindful parenting and goal-setting. In the first visit, caregivers discuss their perceptions and concerns regarding their child and family context. The second visit involves a comprehensive assessment of child and family psychosocial risk and protective factors, including caregiver mental health, relationships and family supports, and video-taped parent-child interactions. In the third visit, caregiver(s) and clinicians engage in a feedback session based on assessment data, using a motivational framework to leverage strengths and motivate change in areas of need. Caregiver and clinician then develop a collaborative menu of services which may include a tailored suite of parenting sessions (using the Everyday Parenting Curriculum [EDP]) linked to the feedback discussion, as well as child, caregiver and/or family mental health or community supports. In the current study, caregivers were offered up to 6 optional EDP sessions as part of their menu of services if specific parenting goals were elicited during the feedback session. For further information, please see Gill et al., 2008^3^ and the following websites: [www.thefamilycheckup.com](http://www.thefamilycheckup.com), and <https://www.cpc.pitt.edu/intervention-models/the-family-check-up/>.

The FCU^®^ has been studied as an early childhood prevention program in two multi-site U.S. RCTs of families of 2 year-old children (*n*=120^4^, *n*=731^5^) enrolled in nutritional programs for low-income families living in urban and rural areas. Follow-up studies into early adolescence have found long-term positive effects on child behaviour problems (including behaviour problems^6^, mood difficulties^7^, substance use^8^) as well as on child obesity^9^ and school readiness^10^, family connectedness to services^11^, caregiver well-being^12^ and positive parenting practices^13^. The FCU® may be flexibly delivered in homes, clinics, community settings or online, and may be delivered incorporating a health maintenance approach with annual check-ups offered during key developmental periods.

Intervention Delivery in Current Study: Unlike standard FCU^®^ delivery, for the purposes of the current study, FCU^®^ assessment data were collected by research staff during the baseline visit and provided to FCU^®^ clinicians for intervention arm participants after randomization. This approach was taken to mitigate potential bias due to caregiver knowledge of treatment allocation prior to completing the baseline study assessment. The FCU^®^ clinician delivered initial interview visit and feedback sessions to caregivers (as well as subsequent EDP sessions).

Treating clinicians recorded the number of sessions of EDP agreed upon with the parent as part of the collaborative “menu” of interventions, and the number of sessions attended (in-home or elsewhere). Over 95% of participants (97.1%) completed all FCU^®^ visits, with 86.5% completing at least one optional EDP visit. FCU^®^ arm participants completed an average of 4 EDP visits post-FCU^®^ feedback, with 44.7% completing the maximum of 6 sessions (Mean EDP session # = 4.1, SD = 2.20; Median = 5).

Clinical Team Training and Supervision: In the current study, 85% of participants were supported by college or undergraduate- level FCU^®^ clinicians, with a Master’s level social worker or PhD Psychology trainee providing the FCU^®^ to the remainder. Participants were assigned to clinicians based on clinician availability and alignment of caregiver-clinician schedules. This reflects the clinical staffing complement of most Ontario child mental health agencies providing care to young children. A psychiatrist (PI) and psychologist (co-PI) provided bi-weekly supervision and individual supervision 1-2x/month supported by videotaped review of sessions.

Clinicians were trained in-person by U.S. trainers over 5 days to deliver the FCU^®^ and linked EDP curriculum, then completed credentialing over 6 months (September 2016 to March 2017). All clinicians were credentialed by U.S. trainers with fidelity coding of 4-6 video-recorded sessions and all sessions were videorecorded throughout study. Fidelity was monitored by study supervisors trained in the COACH rating form, a tool created by program developers to assess competent and adherent delivery of key FCU^®^/EDP conceptual components and therapeutic techniques. Canadian supervisors met 3-4 times/year with a U.S. trainer to conduct random video-recorded reviews of clinician delivery of the model as part of training to become FCU^®^supervisors. McMaster Children’s Hospital in Hamilton, ON then became established as a full-transfer Canadian FCU^®^/EDP delivery site with 2 credentialed supervisors and trainers in August 2019.

Assessment Schedule

At baseline, all participating caregivers provided informed consent and engaged in a baseline assessment of child, caregiver and family characteristics using questionnaire, interview- and observational methods. At the end of the baseline assessment, caregivers were informed in person or by telephone of their allocation to either intervention (FCU^®^) or Community Comparison (CC) group by an unblinded research staff person. For caregivers randomized to intervention, a study clinician contacted them within 1-2 weeks to commence the FCU^®^. Caregivers randomized to the CC arm were provided with a contact list of local community and mental health agencies providing a range of psychosocial supports, e.g., child mental health, housing, food banks, recreation to which they may self-refer.

Randomization was computer-generated by the Juravinski Oncology Clinical Operations Group, in concealed blocks stratified by child sex. Due to the nature of the intervention, participating caregivers were unblinded to allocation arm. Outcome evaluators, assessment staff and data analysts were blinded to allocation arm, with every effort made to maintain blinding throughout conduct of the study. For practical or logistical reasons, the study coordinator, intervention providers and supervisors (including principal investigator) were unblinded to allocation arm, but did not participate in assessment, evaluation or data analysis activities.

Child behaviour problems (primary outcome) and caregiver psychological distress and daily parenting stress (secondary outcomes) were measured at baseline, and then 6- and 12 months later, with additional measures assessed during longer baseline and 12-month visits. Brief telephone check-ins were conducted at 3- and 9- months for participants in both arms to maintain contact, update contact preferences and conduct abbreviated service use questionnaires. Participants received $100 for baseline and 12-month visits, $20 for the 6-month visit and $5 for brief 3- and 9-month check-ins. A data safety and monitoring board (DSMB) met twice yearly to review study processes and adverse events.

**Measures**

*Strengths and Difficulties Questionnaire (SDQ)^14^.* The SDQ is a behavioural screening questionnaire for 2-to-17 year-olds. The questionnaire consists of 25 questions divided into five scales. The prosocial behaviour scale measures strengths, whereas the remaining four evaluate negative behaviours including emotional symptoms, conduct problems, hyperactivity/inattention, and peer relationship problems. The SDQ has demonstrated satisfactory reliability and predictive validity in large samples of European and U.S. preschoolers^15^ as well as convergent and divergent validity with the Child Behaviour Checklist (CBCL) total problems scale and subscores in preschool-aged children^16^.

*Demographics.* The Ontario Child Health Study (OCHS) demographics questionnaire was administered at baseline to assess family socioeconomic status (e.g., gross household income, measured in increments of $10 000, caregiver education and occupation), family structure, child sex at birth and age. We used the Government of Canada definition of LICO for any given year of enrolment of that particular family, which varies by family size and year^17^. For example in 2017, the LICO for a family of 3 in Canada was $37,650.00. If an income range chosen by a family incorporated the LICO for that year, a family was defined as living below the LICO (e.g., a family of 4 people identifying $45,000-$49.0000 income annually, would be defined as living at or below the LICO in 2017, which was $45,712.00).

Concepts of race and ethnicity are nuanced and evolving. In order to describe the reach of study recruitment efforts in comparison to available Statistics Canada regional demographics, primary caregivers reported how they self-identified with specific racialized groups using a specific list from the Canadian 2011 Census National Household Survey, Population Groups and Visible Minorities. Twelve categories were listed alphabetically (e.g., Black, Chinese, Korean, Indigenous, White) as well as “not identified here” (with option to describe) and “prefer not to answer”. Caregivers responded yes/no to any category with which they identified. For purposes of description in this study and in alignment with Statistics Canada measures, respondents were classified as being a member of a visible minority if they identified with at least one category other than “white” in their responses.

*Primary Outcome Measure, Child behaviour problems*: Child Behavior Checklist (CBCL) Externalizing Scale. The CBCL^18^ for ages 1.5 to 5 is a 99-item questionnaire that assesses child emotional and behaviour problems. The 24-item Externalizing Problems Scale was chosen as the primary outcome in line with earlier FCU^®^ evaluations, and because these preschool “acting out” behaviours are highly correlated with concurrent and later childhood mood and behavioural challenges. Caregivers completed items using a 3-point scale (0= *never/not true,* 1= *somewhat/sometimes true,* 2= *very or always true*) at baseline, 6- and 12- months. Items were summed to create a composite score at each time point (T1: α = .90; T2: α = .92; T3: α = .92). In keeping with standard practice in child development research, we used raw CBCL scores to model growth in primary analyses. Raw scores may be converted to normed t-scores, which have established cutoffs indexing clinically severe (65+) and borderline (60-64) scores that were found by measure developers to discriminate between children referred for specialized mental health or special education services from those who were not (normative scores, < 60) .

*Secondary Outcome Measure:* Kessler 6 (K6) Psychological Distress Scale^19^. Caregivers self-reported their levels of psychological distress using the K6 - a brief, 6-item questionnaire measuring anxiety and depression symptoms, which is a valid index of psychiatric disorder and impairment. Cut-off scores of 13+ have been found to index severe depression in the adult Canadian population, with scores of 8-12 representing moderate psychological distress^20^. Items were summed to create a composite score at each time point (T1: α = .85; T2: α = .84; T3: α = .85).

*Covariate: Daily Parenting Stress*. The Parenting Daily Hassles Scale^21,22^ is designed to measure parental perceptions about the minor daily hassles and inconveniences associated with parenting. It comprises 20 items that describe discrete events involving challenging child behaviour or various tasks associated with parenting, with frequency scores used to quantify daily stress in the current study (T1: α = .89; T2: α = .89; T3: α = .91). Good internal consistency and excellent convergent validity have been reported^21^.

Sample size analyses estimated a required sample size of n =210 for the primary outcome based on power analyses estimating a low-moderate effect size of Cohen’s d=0.35 at 12 months, power of 0.80, level of significance 0.05, wave-to-wave correlation of 0.76 between assessments using RMASS2 software. Conservatively estimating an attrition rate of 25% over 12 months yielded an initial recruitment target sample size of n=280^23^.

**Analytic Plan**

All analyses were conducted with Mplus 8.5 using the maximum likelihood robust estimator (MLR). To test the hypothesis that children in the FCU^®^ intervention, relative to those in the CC, would evidence lower behaviour problems scores (primary outcome), caregiver distress, and daily hassles (secondary outcomes) at the 12-month follow up, mean comparisons were conducted using multigroup modeling. We then tested the effect of FCU^®^ intervention on changes in primary (child behaviour problems) and secondary (caregiver distress, parenting stress) outcomes using latent growth curve modeling (LGCM). We first estimated linear and piecewise LGCMs without predictors (unconditional models) to determine the growth function that best characterized the data. In both models, the intercept mean reflected the average level of behaviour problems prior to randomization. In the linear model, the slope mean reflected the estimated rate of linear change across each interval (baseline to 6 months and 6 months to 12 months). In the piecewise growth model, two slope factors were estimated, allowing for the average rate of change to differ between baseline to 6 months (slope 1) and 6 months to 12 months (slope 2). Standard criteria were used to evaluate the fit of the linear model^24^. We then tested a model with predictors (conditional model) by regressing the slope factor(s) onto the dummy-coded treatment status variable (0= CC, 1= FCU^®^). We also included baseline level of caregiver-reported daily parenting stress and child sex as covariates throughout intervention-related analyses, as parenting stress was the only variable found to be significantly different between groups at baseline and randomization was stratified by child sex. In addition to computing end-of-study raw mean differences between FCU^®^ and CC (12-month follow-up) and unstandardized regression coefficients reflecting differences between FCU^®^ and CC on changes in primary and secondary outcomes (growth models), we also calculated Cohen’s *d* effect sizes to describe the magnitude of the standardized mean differences between the treatment and control groups. Following Feingold’s^25^ recommendations for correctly estimating treatment efficacy, ds were calculated by dividing the raw mean difference (12-month follow-up) and regression coefficients (growth models) by the standard deviation of baseline scores. In the growth models, we report effect sizes for differences in interval-to-interval change (i.e., over 6-months) as well as the overall efficacy of the treatment (i.e., over the full 12-months of the study). behaviour problems

Attrition was higher among families with a younger caregiver, lower levels of education, lower household income, and (marginally) higher levels of caregiver-reported psychological distress at T1 (p = .06) and child CBCL externalizing scores at T1 (p = .10). This suggested that longitudinal attrition was partly accounted for by study variables and, as such, could reasonably be handled under a missing-at-random (MAR) assumption using full-information maximum likelihood (FIML) according to best practice principles. We therefore retained the full sample for all analyses (*N* = 206). To support the estimation of missing data under the MAR assumption, we included correlates of missingness as auxiliary variables in all models in keeping with recommended practice^26^.

**Adverse Events and Study Monitoring**

As a psychological and behavioural intervention, the current study involved relatively minimal risk. Adverse events (AEs) include any untoward health-related outcome that occurs during the study, regardless of relatedness to the study intervention. Anticipated adverse events relevant to this study included those related to parent, child and clinician safety and psychological well-being, as well as impactful family stressors (e.g., marital separation, caregiver incarceration, school suspensions, caregiver suicidality). Adverse events were not systematically elicited by study team members but were recorded if they were reported by caregivers, children, or others (e.g., child protection worker, school staff) to study staff. Anticipated adverse events that were not serious were discussed as needed by research staff and FCU^®^ clinicians with the primary investigator or back-up, if the nature of the adverse event was considered to signal unresolved risk to parent, child or clinician.

A protocol for serious adverse events (e.g., hospitalization, death of a participant) was developed, which included notifying the principal investigator immediately as well as the research ethics board within 24 hours. Reports to child protection agencies were noted separately from adverse events, after guidance from the Data and Safety Monitoring Board (DSMB), due to higher likelihood of incidence within the intervention arm related to surveillance effects (i.e., higher number of interactions). There were no significant differences between numbers of adverse events between intervention arms, including 4 serious adverse events deemed unrelated to study participation (2 in each arm, caregiver and child hospitalizations due to accident or illness).

A DSMB approved the study protocol and met twice a year to review recruitment rates and issues, review study risk mitigation strategies and adverse events and determine if any additional information or protocol changes were needed. The board deemed that stoppage rules were not required given the low-risk nature of the trial.

**Results**

Mean child behaviour problems and K6 Caregiver distress scores across time points are summarized in Table 3, main manuscript. At baseline, 48.5% of the sample met cut-off criteria for clinical levels (90th percentile) of externalizing problems. The average CBCL score was approximately 1 SD above the population average for preschool-aged children^18^. Among primary caregivers, 49% endorsed at least moderate psychological distress. Recruitment for the trial was stopped March 31, 2019 prior to reaching the anticipated sample size of n=280 to ensure complete 12-month follow-up prior to completion of funding period. Follow-up assessments were completed by June 30, 2020. Only 103 FCU^®^ participants were included in analyses because one child was enrolled twice unbeknownst to study team, due to changes in custodial caregivers, after which the second custodial caregiver enrolled in the study independently. Data were included for the second caregiver, who completed all research timepoints, with sensitivity analyses demonstrating that there were no meaningful differences found by including the first caregiver instead (see Figure 1, manuscript).

Growth Models

Child behaviour problems as measured using the CBCL Externalizing Problems Score. The linear LGCM for behaviour problemsprovided a poor fit to the data, χ2 (3) = 32.81, p < .001, RMSEA= .22, CFI= .91, SRMR= .08. Estimates from the piecewise growth model indicated that rates of change in behaviour problems differed from baseline and 6-month, and 6-month to 12-month follow-up (see Table 3 and Figures 2(a-c). Average levels of child behaviour problems symptoms declined from baseline to 6-month follow up (slope 1), whereas no significant mean level change was observed between 6- and 12-month follow up (slope 2). We then tested a conditional model with intervention condition, daily hassles, and child sex included as predictors of behaviour problems slopes. A model with the predictor effects constrained to equality across the two slopes provided an excellent fit to the data, χ^2^ (3) = 1.57, *p* =.67, RMSEA= .00, CFI= .99, SRMR= .02, and allowing the intervention effects to differ across the slopes did not significantly improve model fit, Δχ^2^ (1) = 0.08, *p* = .77. Children whose parents participated in the FCU^®^ showed faster declines in behaviour problems (interval-to-interval change *d* = .19; overall treatment efficacy *d* = .38).

Caregiver Psychological Distress. The unconditional linear model for caregiver distress provided acceptable fit to the data, χ^2^ (3) = 3.18, *p* =.37, RMSEA= .02, CFI= .99, SRMR= .03; average levels of caregiver distress linearly declined over time. Results from the conditional model indicated that caregivers in the FCU^®^ did not decline at a significantly different rate from caregivers in the CC (interval-to-interval change *d* = .08; overall treatment efficacy *d* = .16).

Caregiver Daily Hassles. The unconditional linear model for daily hassles provided a poor fit to the data, χ^2^ (3) = 19.66, *p* < .001, RMSEA= .16, CFI= .93, SRMR= .06. Estimates based on the piecewise model indicated that daily hassles declined from baseline to 6-month, whereas no significant mean level decline was observed between the 6- and 12-month follow-ups. Rates of change in daily hassles did not differ between caregivers in the FCU^®^ and CC groups (interval-to-interval change *d* = .02; overall treatment efficacy *d* = .04).

Table 1. Longitudinal Attrition Analyses Comparing Baseline Characteristics for Participants with Complete Data (n = 176) vs. Dropouts (n = 30)

Table 2. Full correlation table for all variables

Table 3. Correlation table for primary and secondary outcomes

Table 4. Correlation table for primary and secondary outcomes, by condition

Table 5. Sensitivity analyses for assessments conducted prior to vs. during COVID19 pandemic lockdown period.

Table 1

*Longitudinal Attrition Analyses Comparing Baseline Characteristics for Participants with Complete Data (n = 176) vs. Dropouts (n = 30)*

|  | Complete | | Dropouts | |  |  |
| --- | --- | --- | --- | --- | --- | --- |
|  | M / % | SD | M / % | SD | Test statistic | *p* value |
| Study condition (% FCU^®^) ^a^ | 51% |  | 46% |  | 0.3 | .6 |
| Child sex (% female) ^a^ | 44% |  | 35% |  | 1.1 | .3 |
| Parent sex (% female) ^a^ | 98% |  | 97% |  | 0.1 | .9 |
| Child age ^b^ | 3.40 | 0.8 | 3.5 | 1.0 | 0.3 | .6 |
| Parent age ^b c^ | 34.13 | 5.3 | 31.4 | 6.09 | 7.5 | .007 |
| Biological parent (% yes)^a^ | 97% |  | 95% |  | 0.6 | .5 |
| Parent marital status (% marred) ^a^ | 77% |  | 51% |  | 9.5 | .002 |
| Child minority status (% minority) ^a^ | 28% |  | 35% |  | 0.7 | .4 |
| Parent minority status (% minority) ^a^ | 18% |  | 22% |  | 0.2 | .7 |
| Parent education ^b^ | 6.1 | 2.4 | 4.6 | 2.3 | 10.78 | .001 |
| Household income ^b^ | 12.8 | 6.8 | 9.6 | 7.0 | 5.73 | .02 |
| Financial stress ^b^ | 2.4 | 0.9 | 2.8 | 0.9 | 5.40 | .02 |
| Parental psychological distress ^b^ | 7.5 | 4.6 | 9.2 | 5.8 | 3.51 | .06 |
| Parent daily hassles ^b^ | 2.5 | 0.5 | 2.5 | 0.5 | 1.59 | .2 |
| Child externalizing problems ^b^ | 24.9 | 8.8 | 27.6 | 9.4 | 2.80 | .1 |

*Note.* Test statistics represent chi-square values (for dichotomous variables) or F values (for continuous variables)

^a^ Dichotomously scored variable. ^b^ Continuously scored variable. ^c^ Younger parents more likely to drop out earlier.

Table 2. Full bivariate correlation table for all variables

|  |  | 1. | 2. | 3. | 4. | 5. | 6. | 7. | 8. | 9. | 10. | 11. | 12. | 13. | 14. | 15. | 16. | 17. | 18. | 19. |
| --- | --- | --- | --- | --- | --- | --- | --- | --- | --- | --- | --- | --- | --- | --- | --- | --- | --- | --- | --- | --- |
| 1. | Condition (FCU^®^) | – |  |  |  |  |  |  |  |  |  |  |  |  |  |  |  |  |  |  |
| 2. | T1 externalizing | -.04 | – |  |  |  |  |  |  |  |  |  |  |  |  |  |  |  |  |  |
| 3. | T2 externalizing | -.10 | .71^*^ | – |  |  |  |  |  |  |  |  |  |  |  |  |  |  |  |  |
| 4. | T3 externalizing | -.20^*^ | .68^*^ | .78^*^ | – |  |  |  |  |  |  |  |  |  |  |  |  |  |  |  |
| 5. | T1 K6 | .01 | .40^*^ | .29^*^ | .29^*^ | – |  |  |  |  |  |  |  |  |  |  |  |  |  |  |
| 6. | T2 K6 | -.08 | .31^*^ | .35^*^ | .28^*^ | .70^*^ | – |  |  |  |  |  |  |  |  |  |  |  |  |  |
| 7. | T3 K6 | -.06 | .35^*^ | .43^*^ | .44^*^ | .59^*^ | .71^*^ | – |  |  |  |  |  |  |  |  |  |  |  |  |
| 8. | Child sex (female) | 0 | -.02 | -.03 | .00 | .08 | .07 | .09 | – |  |  |  |  |  |  |  |  |  |  |  |
| 9. | Parent sex (female) | -0.10 | .08 | .03 | .15^*^ | .00 | -.01 | .02 | .07 | – |  |  |  |  |  |  |  |  |  |  |
| 10. | Child age | -.04 | -.04 | -.07 | .07 | -.01 | .01 | .03 | -.12^†^ | -.23^*^ | – |  |  |  |  |  |  |  |  |  |
| 11. | Parent age | -.02 | -.05 | .01 | -.02 | -.12 | -.05 | -.11 | -.04 | -.07 | .13^†^ | – |  |  |  |  |  |  |  |  |
| 12. | Bio parent (yes) | -.08 | -.14^†^ | -.09 | -.10 | -.03 | -.02 | .05 | -.06 | -.03 | -.01 | -.19^*^ | – |  |  |  |  |  |  |  |
| 13. | Marital status (yes) | -.01 | .01 | -.04 | .01 | -.15^*^ | -.26^*^ | -.15^*^ | .11 | .25^*^ | -.15 | .24^*^ | .00 | – |  |  |  |  |  |  |
| 14. | Child minority (non-White) | -.01 | .09 | .14 | .12^†^ | .11 | .14^*^ | .15^*^ | .06 | -.11 | .07 | .07 | -.05 | -.18^*^ | – |  |  |  |  |  |
| 15. | Parent minority (non-White) | .01 | .03 | -.01 | .04 | .12 | .07 | .11 | .11 | -.17^†^ | .13^†^ | .00 | -.05 | -.08 | .66^*^ | – |  |  |  |  |
| 16. | Parent education | -.02 | -.12^†^ | -.18^*^ | -.10 | -.20^*^ | -.16^*^ | -.27^*^ | -.01 | .14^*^ | -.09 | .34^*^ | -.04 | .37^*^ | -.11 | -.05 | – |  |  |  |
| 17. | Household income | -.06 | -.02 | -.10 | -.08 | -.32^*^ | -.28^*^ | -.25^*^ | .03 | .04 | -.09 | .35^*^ | -.14^*^ | .48^*^ | -.17^*^ | -.11 | .61^*^ | – |  |  |
| 18. | T1 financial stress | .05 | .18 | .26 | .19 | .41^*^ | .38^*^ | .40^*^ | -.02 | -.06 | .02 | -.18^*^ | .10 | -.34^*^ | .16^*^ | .07 | -.32^*^ | -.55^*^ | – |  |
| 19. | T1 daily hassles | -.15 | .59^*^ | .49^*^ | .49^*^ | .38^*^ | .27^*^ | .34^*^ | -.05 | .15^*^ | -.05 | .10 | -.10^†^ | .07 | .07 | .00 | .04 | -.01 | .19^*^ | ­– |

*Note. N* = 206; T= time; externalizing= CBCL externalizing scores; K6= parent psychological distress scores from the Kessler 6 scale; Bio= biological;

† *p* ≤ .10. * *p* ≤ .05

Table 3 Correlation table for primary and secondary outcomes

|  |  |  | Primary Outcome | | |  | Secondary Outcome | | |
| --- | --- | --- | --- | --- | --- | --- | --- | --- | --- |
|  | Condition (FCU^®^) |  | T1 Exter | T2 Exter | T3 Exter |  | T1 K6 | T2 K6 | T3 K6 |
| Condition (FCU^®^) | – |  |  |  |  |  |  |  |  |
| T1 externalizing | -.04 |  | – |  |  |  |  |  |  |
| T2 externalizing | -.10 |  | .71^*^ | – |  |  |  |  |  |
| T3 externalizing | -.20^*^ |  | .68^*^ | .78^*^ | – |  |  |  |  |
| T1 K6 | .01 |  | .40^*^ | .29^*^ | .29^*^ |  | – |  |  |
| T2 K6 | -.08 |  | .31^*^ | .35^*^ | .28^*^ |  | .70^*^ | – |  |
| T3 K6 | -.06 |  | .35^*^ | .43^*^ | .44^*^ |  | .59^*^ | .71^*^ | – |
| Child sex (female) | 0 |  | -.02 | -.03 | 0 |  | .08 | .07 | .09 |
| Parent sex (female) | -.10 |  | .08 | .03 | .15^*^ |  | 0 | -.01 | .02 |
| Child age | -.04 |  | -.04 | -.07 | .07 |  | -.01 | .01 | .03 |
| Parent age | -.02 |  | -.05 | .01 | -.02 |  | -.12 | -.05 | -.11 |
| Bio parent (yes) | -.08 |  | -.14^†^ | -.09 | -.10 |  | -.03 | -.02 | .05 |
| Marital status (yes) | -.01 |  | .01 | -.04 | .01 |  | -.15^*^ | -.26^*^ | -.15^*^ |
| Child minority (non-White) | -.01 |  | .09 | .14 | .12^†^ |  | .11 | .14^*^ | .15^*^ |
| Parent minority (non-White) | .01 |  | .03 | -.01 | .04 |  | .12 | .07 | .11 |
| Parent education | -.02 |  | -.12^†^ | -.18^*^ | -.10 |  | -.20^*^ | -.16^*^ | -.27^*^ |
| Household income | -.06 |  | -.02 | -.10 | -.08 |  | -.32^*^ | -.28^*^ | -.25^*^ |
| T1 financial stress | .05 |  | .18 | .26 | .19^*^ |  | .41^*^ | .38^*^ | .40^*^ |
| T1 daily hassles | -.15 |  | .59^*^ | .49^*^ | .49^*^ |  | .38^*^ | .27^*^ | .34^*^ |

*Note. N* = 206; T= time; Exter= child CBCL externalizing scores; K6= parent Kessler 6 psychological distress scores; Bio= biological.

† *p* ≤ .10. * *p* ≤ .05

Table 4. Correlation table for primary and secondary outcomes, by condition

|  | Community Control | | | | | |  | FCU^®^ | | | | | |
| --- | --- | --- | --- | --- | --- | --- | --- | --- | --- | --- | --- | --- | --- |
|  | T1  Exter | T2 Exter | T3  Exter | T1  K6 | T2  K6 | T3  K6 |  | T1  Exter | T2 Exter | T3  Exter | T1  K6 | T2  K6 | T3  K6 |
| T1 externalizing | – |  |  |  |  |  |  |  |  |  |  |  |  |
| T2 externalizing | .71^*^ | – |  |  |  |  |  | .72^*^ |  |  |  |  |  |
| T3 externalizing | .66^*^ | .70^*^ | – |  |  |  |  | .70^*^ | .80^*^ |  |  |  |  |
| T1 K6 | .26^*^ | .20^†^ | .27^*^ | – |  |  |  | .51^*^ | .36^*^ | .33^*^ |  |  |  |
| T2 K6 | .29^*^ | .28^*^ | .21^†^ | .72^*^ | – |  |  | .34^*^ | .43^*^ | .32^*^ | .69^*^ |  |  |
| T3 K6 | .32^*^ | .29^*^ | .39^*^ | .75^*^ | .71^*^ | – |  | .38^*^ | .57^*^ | .48^*^ | .47^*^ | .71^*^ |  |
| Child sex (female) | -.08 | -.08 | -.17^†^ | .09 | .04 | .05 |  | .04 | .02 | .14 | .07 | .13 | .10 |
| Parent sex (female) | .07 | .05 | .19^*^ | .14^*^ | .15^*^ | .16^*^ |  | .09 | .00 | .11 | -.07 | -.13 | -.06 |
| Child age | .02 | -.13 | .15 | .06 | -.07 | .08 |  | -.10 | -.04 | .01 | -.07 | .09 | -.03 |
| Parent age | .04 | .14 | .02 | -.08 | -.01 | -.06 |  | -.14 | -.14 | -.06 | -.17 | -.12 | -.17 |
| Bio parent (yes) | -.16^*^ | -.11 | -.07 | -.04 | -.05 | -.05 |  | -.15 | -.09 | -.18^†^ | -.03 | -.02 | .11 |
| Marital status (yes) | -.11 | -.10 | -.09 | -.30^*^ | -.29^*^ | -.19^†^ |  | .12 | .01 | .09 | -.02 | -.25^*^ | -.15 |
| Child minority (non-White) | .12 | .26^*^ | .10 | .08 | .12 | .10 |  | .06 | .06 | .14 | .14 | .17 | .23^*^ |
| Parent minority (non-White) | -.07 | -.03 | -.02 | .00 | -.02 | .05 |  | .11 | .02 | .10 | .23^†^ | .21^†^ | .16 |
| Parent education | -.10 | -.16 | -.11 | -.24^*^ | -.17^*^ | -.21^*^ |  | -.13 | -.22^*^ | -.10 | -.17 | -.20^*^ | -.33 |
| Household income | -.01 | -.04 | -.06 | -.39^*^ | -.30^*^ | -.25^*^ |  | -.05 | -.17 | -.10 | -.25^*^ | -.29^*^ | -.26^*^ |
| T1 financial stress | .08 | .16^†^ | .13 | .40^*^ | .31^*^ | .38^*^ |  | .27^*^ | .35^*^ | .27^*^ | .43^*^ | .47^*^ | .45^*^ |
| T1 daily hassles | .52^*^ | .51^*^ | .43^*^ | .29^*^ | .23^*^ | .36^*^ |  | .66^*^ | .46^*^ | .51^*^ | .48^*^ | .30^*^ | .31^*^ |

*Note. N* = 206; T= time; Exter= behaviour problems as measured using child CBCL externalizing scores; K6= parent Kessler 6 psychological distress scores; Bio= biological

† *p* ≤ .10. * *p* ≤ .05

Table 5. Sensitivity analyses: assessments conducted prior to vs. during COVID19 pandemic lockdown period.

|  |  | Child Behavior Problems | | |  | Caregiver Distress | | |  | Daily Parenting Stress | | |
| --- | --- | --- | --- | --- | --- | --- | --- | --- | --- | --- | --- | --- |
|  |  | Estimate | *p* ≤ | Overall treatment efficacy (d) |  | Estimate | *p* ≤ | Overall treatment efficacy (d) |  | Estimate | *p* ≤ | Overall treatment efficacy (d) |
|  |  |  |  |  |  |  |  |  |  |  |  |  |
| Model 1 | |  |  |  |  |  |  |  |  |  |  |  |
|  | Slope 1 intercept | -4.47 | .001 |  |  | -0.25 | .19 |  |  | -0.21 | .001 |  |
|  | Slope 2 intercept | 0.36 | .91 |  |  | -- | -- |  |  | 0.02 | .57 |  |
|  | Condition 🡪 slope | -1.69 | .003 | -.38 |  | -0.35 | .28 | -.17 |  | 0.03 | .78 | .05 |
| Model 2 | |  |  |  |  |  |  |  |  |  |  |  |
|  | Slope 1 intercept | -4.49 | .001 |  |  | -0.38 | .09 |  |  | -0.21 | .001 |  |
|  | Slope 2 intercept | -0.07 | .91 |  |  | -- | -- |  |  | 0.02 | .64 |  |
|  | Condition 🡪 slope | -1.68 | .003 | -.38 |  | -0.37 | .30 | -.17 |  | 0.01 | .71 | .05 |
| Model 3 | |  |  |  |  |  |  |  |  |  |  |  |
|  | Slope 1 intercept | -4.61 | .001 |  |  | -0.22 | .23 |  |  | -0.21 | .001 |  |
|  | Slope 2 intercept | 0.08 | .89 |  |  | -- | -- |  |  | 0.03 | .42 |  |
|  | Condition 🡪 slope | -1.69 | .001 | -.38 |  | -0.54 | .09 | -.25 |  | 0.00 | .90 | .02 |
|  | Covid status | 2.47 | .10 |  |  | 1.55 | .014 |  |  | -0.02 | .85 |  |

Model 1= original results reported in manuscript; Model 2= model treating data collected during Covid as missing; Model 3= model adjusting for the effect of covid status on 12-month scores (0= *completed before Covid*, 1= *completed during Covid*); Estimates for predictors represent unstandardized regression coefficients.

References

1. City of Hamilton. (n.d). *Child and Youth Health Atlas*. Retrieved 18 Jul 2022 from https://www.hamilton.ca/people-programs/public-health/healthy-schools/child-and-youth-health-atlas

2. Siddiqua A, Duku E, Georgiades K, Mesterman R, Janus M. (2012). Association between neighbourhood socioeconomic status and developmental vulnerability of kindergarten children with Autism Spectrum Disorder: A population level study. *SSM Popul Health*, *12*:100662.

3. Gill AM, Hyde LW, Shaw DS, Dishion TJ, Wilson MN. (2008). The Family Check-Up in early childhood: A case study of intervention process and change. *Journal of Clinical Child and Adolescent Psychology*, *37*(4):893-904.

4. Dishion, T. J., Shaw, D., Connell, A., Gardner, F., Weaver, C., & Wilson, M. (2008). The Family Check-Up with high-risk indigent families: Preventing problem behavior by increasing parents’ positive behavior support in early childhood. *Child Development, 79*(5):1395–1414.

5. Shaw DS, Dishion TJ, Supplee L, Gardner F, Arnds K. (2006). Randomized trial of a family-centered approach to the prevention of early conduct problems: 2-year effects of the Family Check-Up in early childhood. *Journal of Consulting and Clinical Psychology*, *74*(1):1-9.

6. Shaw DS, Sitnick SL, Brennan LM, Choe DE, Dishion TJ, Wilson MN, et al. (2016). The long-term effectiveness of the Family Check-Up on school-age conduct problems: Moderation by neighborhood deprivation. *Development and Psychopathology, 28*(4pt2):1471-1486.

7. Connell AM, Shaw D, Wilson M, Danzo S, Weaver-Krug C, Lemery-Chalfant K, et al. (2019). Indirect effects of the early childhood Family Check-Up on adolescent suicide risk: The mediating role of inhibitory control. *Development and Psychopathology*, *31*(5):1901-1910.

8. Connell AM, Dishion TJ, Deater-Deckard K. (2006). Variable- and person-centered approaches to the analysis of early adolescent substance Use: Linking peer, family, and intervention effects with developmental trajectories. *Merrill-Palmer Quarterly: Journal of Developmental Psychology*, *52*(3):421-448.

9. Smith JD, Montano Z, Dishion TJ, Shaw DS, Wilson MN. (2015). Preventing weight gain and obesity: Indirect effects of the Family Check-Up in early childhood. *Prevention Science,* 16(3):408-419.

10. Lunkenheimer ES, Dishion TJ, Shaw DS, Connell AM, Gardner F, Wilson MN, et al. (2008). Collateral benefits of the Family Check-Up on early childhood school readiness: Indirect effects of parents' positive behavior support. *Developmental Psychology, 44(*6):1737-1752.

11. Leijten P, Shaw DS, Gardner F, Wilson MN, Matthys W, Dishion TJ. (2015). The Family Check-Up and service use in high-risk families of young children: A prevention strategy with a bridge to community-based treatment. *Prevention Science*, *16*(3):397-406.

12. Reuben JD, Shaw DS, Brennan LM, Dishion TJ, Wilson MN. (2015). A family-based intervention for improving children's emotional problems through effects on maternal depressive symptoms. *Journal of Consulting and Clinical Psychology*, *83*(6):1142-1148.

13. Sitnick SL, Shaw DS, Gill A, Dishion T, Winter C, Waller R, et al. (2015). Parenting and the Family Check-Up: Changes in observed parent-child interaction following early childhood intervention. *Journal of Clinical Child and Adolescent Psychology, 44*(6):970-984.

14. Goodman R. (2001). Psychometric properties of the Strengths and Difficulties Questionnaire. *Journal of the American Academy of Child and Adolescent Psychiatry*, *40*(11):1337-1345.

15. D'Souza S, Waldie KE, Peterson ER, Underwood L, Morton SM. (2017). Psychometric properties and normative data for the preschool Strengths and Difficulties Questionnaire in two-year-old children. *Journal of Abnormal Child Psychology,45*(2):345-357.

16. Theunissen MH, Vogels AG, de Wolff MS, Reijneveld SA. (2013). Characteristics of the Strengths and Difficulties Questionnaire in preschool children. *Pediatrics*, *131*(2):e446-54.

17. Statistics Canada. Table 11-10-0241-01. (2022). Low income cut-offs (LICOs) before and after tax by community size and family size, in current dollars.

18. Achenbach TM, Rescorla L. (2001). *Manual for the ASEBA school-age forms & profiles: An integrated system of multi-informant assessment*: ASEBA Burlington, VT.

19. Kessler RC, Barker PR, Colpe LJ, Epstein JF, Gfroerer JC, Hiripi E, et al. (2003). Screening for serious mental illness in the general population. *Archives of General Psychiatry*, *60*(2):184-189.

20. Chiu M, Lebenbaum M, Cheng J, de Oliveira C, Kurdyak P. (2017). The direct healthcare costs associated with psychological distress and major depression: A population-based cohort study in Ontario, Canada. *PLoS One*, *12*(9):e0184268.

21. Crnic KA, Booth CL. (1991). Mothers' and fathers' perceptions of daily hassles of parenting across early childhood. *Journal of Marriage and Family*, *53*(4):1042-1050.

22. Crnic KA, Greenberg MT. (1990). Minor parenting stresses with young children. *Child Development, 61*(5):1628-1637.

23. Hedeker D, Gibbons RD, Waternaux C. (1999). Sample size estimation for longitudinal designs with attrition: Comparing time-related contrasts between two groups. *Journal of Educational and Behavioral Statistics, 24*(1):70-93.

24. Curran PJ, Obeidat K, Losardo D. (2010). Twelve frequently asked questions about growth curve modeling. *Journal of Cognition and Development, 11*(2):121-136.

25. Feingold, A. (2009). Effect sizes for growth-modeling analysis for controlled clinical trials in the same metric as for classical analysis. *Psychological Methods, 14(*1):43-53.

26. Rioux C, Little TD. (2021). Missing data treatments in intervention studies: What was, what is, and what should be. *International Journal of Behavioral Development, 45*(1):51-58.
